# Molecular Biology of Human Fertility: Stepping towards a Tailored Approach

**DOI:** 10.3390/ijms23147517

**Published:** 2022-07-07

**Authors:** Antonio Simone Laganà, Stefano Uccella, Vito Chiantera, Simone Garzon

**Affiliations:** 1 Unit of Gynecologic Oncology, ARNAS “Civico–Di Cristina–Benfratelli”, Department of Health Promotion, Mother and Child Care, Internal Medicine and Medical Specialties (PROMISE), University of Palermo, 90127 Palermo, Italy; vito.chiantera@unipa.it; 2 Department of Obstetrics and Gynecology, AOUI Verona, University of Verona, 37126 Verona, Italy; stefano.uccella@univr.it (S.U.); simone.garzon@univr.it (S.G.)

Clinical pregnancies and live births result from a complex interaction of molecular pathways at the level of the female and male gametes during their development and interaction for fecundation, and the subsequent development of the embryo before, during, and after implantation. All these mechanisms interact with the surrounding environment present at the level of the gonads and male and female bodies, and later, at the level of the female genital tract: from tubes to the endometrium and uterus. To date, the knowledge of this molecular biology underlining human fertility has been paramount to understanding physiology and pathology, with the ultimate objective of successfully characterizing and treating female and male infertility [1]. In this scenario, the aim of the Special Issue “Molecular Biology of Human Fertility” was to collect cutting-edge research and to summarize the most robust pieces of evidence on the molecular aspects of the complex mechanisms of human fertility.

In this Special Issue, the study by Olszewska et al. [2] allowed us to obtain reliable and novel data about the role of epigenetic changes in male infertility. Indeed, this study aimed to determine the relationship between methylation (5mC) and hydroxymethylation (5hmC) in sperm DNA with respect to sperm chromatin protamination in three subpopulations of fertile normozoospermic controls and infertile patients with oligo-/oligoasthenozoospermia. For the first time, a sequential staining protocol was applied, which allowed the researchers to analyze 5mC/5hmC levels by immunofluorescence staining, with a previously determined chromatin protamination status (aniline blue staining), using the same spermatozoa. Based on their data analyses, the authors found a relationship between 5mC/5hmC and sperm motility/morphology: in particular, the linkage between chromatin protamination and 5mC/5hmC levels in control males disappeared in patients with deteriorated semen parameters, suggesting that the 5mC/5hmC status of sperm DNA according to sperm chromatin integrity provides evidence of correct spermatogenesis. In this field, accumulating evidence indicates that regulation of genetic stability in germ cells plays a pivotal role in stem cell maintenance and transposon repression in the human germline, allowing subsequent development and maturation. From this perspective, Giebler et al. [3] analyzed how PIWI-LIKE 1-4 mRNA expression in ejaculated spermatozoa predicts outcomes of assisted reproductive techniques (ART), evaluating swim-up spermatozoa used for fertilization from 160 in vitro fertilization (IVF) or intracytoplasmic sperm injection (ICSI) cycles. Of note, PIWI-LIKE 1 and 2 transcript levels in the spermatozoa of the swim-up fraction were positively correlated with each other; moreover, lower PIWI-LIKE 2 mRNA levels, as well as lower PIWI-LIKE 1 mRNA levels, in these spermatozoa were positively associated with a fertilization rate of ≥50% in the ART cycles. Considering the key role of the morphology of male gametes for fertility, the study by Sáez-Espinosa et al. [4] provided new insights into globozoospermia, which could have potential implications for improving sperm selection methods for ARTs. Indeed, using a combination of field-emission scanning electron microscopy (FE-SEM) and transmission electron microscopy (TEM), they identified and correlated eight morphological patterns with both types of microscopies; in addition, using fluorescent microscopy, they found that most of the sperm showed tubulin in the terminal piece of the flagellum, and less than 1% displayed tyrosine phosphorylation in the flagellum.

The most relevant pieces of evidence about the transcription factor p63, pivotal in regulating maternal reproduction and genomic integrity as well as epidermal development, were collected in the review by Luan et al. [5]. In particular, TAp63 expression turns on in the nuclei of primordial germ cells in females and is maintained mainly in the oocyte nuclei of immature follicles. Accumulating data suggest that TP63 mutations are connected with female infertility, including isolated premature ovarian insufficiency (POI).

One of the most common diseases associated with female infertility is endometriosis [6], although the exact causal mechanism is uncertain and reliable markers are still lacking [7]. For this reason, we were pleased to accept for publication the study by Lis-Kuberka and collaborators [8], who used ELISA and SDS-agarose immunoblotting to determine the presence and concentration of fibronectin and the occurrence of soluble FN–fibrin complexes, respectively, in the blood plasma of women with endometriosis, of those with fertility disorders, and of a healthy group. Of note, they found that the concentration of fibronectin in the blood plasma of women with endometriosis and fertility disorders was significantly higher than that of the healthy group. On that basis, the presence of FN–fibrin complexes with a molecular mass higher than 1300 kDa in women with endometriosis and infertility and the complete absence of these complexes in healthy women may indicate an increased and chronic activation of coagulation mechanisms in these patients, confirming previous findings [9].

Besides male and female infertility factors, which are not mutually exclusive to each other, the events at the endometrial–embryonal interface are of paramount importance. Indeed, failure of these events can lead to recurrent implantation failure (RIF) and recurrent pregnancy loss (RPL), regardless of the use of ARTs. Calcium signaling pathways are known to be of vital importance to embryo implantation and pregnancy establishment; anterior gradient protein 3 (AGR3) and S100 calcium-binding protein P (S100P) are involved in these pathways, and they were elegantly investigated by Tempest et al. [10]. In this study, the authors found significantly higher AGR3 and S100P immunostaining in the ciliated cells of the luminal epithelium of women with recurrent reproductive failure compared with parous women, suggesting an aberrant subcellular location-associated pathophysiology for these conditions.

When fertility ends, it is time to recall whether some molecular changes may increase the risk of cancer [11]. In this regard, Kawahara and colleagues [12] showed, for the first time, that Cyclin-E1 (CCNE1) is a synthetic lethal target gene to ARID1A-mutated ovarian clear cell carcinoma. Targeting this gene may represent a putative, novel, anticancer strategy in ovarian cancer treatment, besides currently available approaches [13].

We believe that the studies published in the Special Issue “Molecular Biology of Human Fertility” provide cutting-edge research and summarize the most robust pieces of evidence on the molecular aspects of the complex mechanisms of human fertility, representing a starting point for future research on such a relevant aspect of human life.
